# Education Level Among Patients with Major Limb Amputation

**DOI:** 10.7759/cureus.7673

**Published:** 2020-04-14

**Authors:** Ivan Chernev, Alexandra Chernev

**Affiliations:** 1 Orthopedics, Medical University of South Carolina (MUSC) Health Florence Medical Center, Florence, USA; 2 Physical Medicine and Rehabilitation, Palmetto Amputee Network, Florence, USA

**Keywords:** education, major limb amputation, limb loss, socioeconomic, rehabilitation

## Abstract

Introduction

Despite all the advances in medicine and attempts to delay and prevent amputations, the number of amputations remains high. The state of South Carolina has one of the highest rates of major limb amputation in the country, with Florence and neighboring counties particularly affected. Education level has been associated with an increased number of amputations and worse outcomes post-amputation. The aim of this study was to investigate the education level among patients with major upper and lower limb amputation within the Florence and neighboring counties, which is a part of the Pee Dee region of the state of South Carolina.

Materials and methods

This is a retrospective chart review study conducted at the outpatient hospital-based Physical Medicine and Rehabilitation clinic. All consecutive amputee patients visiting our outpatient amputee clinic from January 2018 to January 2019 and who met the study inclusion criteria were included in the study. The main outcome measure was the education level.

Results

Of the sample, 26% had below high school education, 33.8% had high school education, 14.2% had some college education, 19.7% had a college education, and 6.3% had an advanced college degree.

Conclusions

Patients with major limb amputation have a lower education level compared to the general population. Lower education level as a part of the broader and more complex socioeconomic status may be a possible barrier in the process of post-amputation rehabilitation and long-term care of patients with major limb amputation.

## Introduction

Limb loss is a major and a life-changing event leading to permanent disability, significant functional impairment, and decreased quality of life [1,2]. Each year, an estimated 185,000 people undergo amputation in the United States, with the overall number of amputations being performed increasing [3,4]. Furthermore, nearly two million individuals have some level of limb amputation, with most amputations affecting the lower limb [3,5]. In addition, the state of South Carolina has one of the highest rates of major limb amputation in the country, with Florence and neighboring counties particularly affected [6,7]. Goodney et al. reported an amputation rate of more than 23 per 10,000 Medicare patients with the peripheral arterial disease (PAD) in Florence, South Carolina [6]. Certain factors, such as age, gender, race, co-existing diseases, smoking, and socioeconomic status, among others, have been associated with higher rates of amputation [6,8-12]. For example, Ciocan et al. identified age as a risk factor for amputation [8]. Arya et al. found the race to be independently predictive of major limb amputation in patients with PAD [11]. Ferguson et al., Arya et al., Stevens et al., and Goodney et al. all showed a positive association between low socioeconomic status and rates of major limb amputation [6,10-12].

Despite all the advances in medicine and attempts to delay and prevent amputations, the number of amputations remains high. With the increasing prevalence of amputations, it is equally important to manage patients' post-amputation. Achieving independence and regaining the ability to walk can have a significant impact on the amputee's quality of life and reintegration in the community. Furthermore, maintaining mobility, functional level, and preventing complications from prosthetic use is a critical part of the long-term management of patients with major limb amputation. Variables such as age, etiology of amputation, level of amputation, bilateral amputation, body mass index, phantom and residual limb pain, co-morbidities, marital status, psychosocial support, and access to medical care and rehabilitation services can affect adjustment to amputation, post-amputation and prosthetic rehabilitation, and long-term prosthetic use [4,5,13-15]. Education level has also been previously cited to affect different outcome measures and mortality after amputation. Nunes et al. found that patients with low education level showed lower rates of adaptation to prosthesis [14]. In a review of the literature, Darter et al. found that education level was associated with return to work rates [5]. Amputees with higher education level had more job opportunities and more flexibility in their work schedules [5]. Corey et al. showed increased five-year mortality for patients who did not complete high school education compared to those who completed high school education [16]. They also found that patients who completed high school education were more likely to ambulate with a prosthesis following amputation [16]. As education may affect different aspects of post-amputation rehabilitation, understanding regional population-based education level differences among patients with major limb amputation will allow proper strategy and utilization of resources in the management of this less fortunate population. Therefore, the aim of this study was to investigate the education level among patients with major upper and lower limb amputation within the Florence and neighboring counties, which is a part of the Pee Dee region of South Carolina.

## Materials and methods

This was a retrospective chart review study. The study was approved by the institutional review board. Data were collected from the electronic medical records of all patients with major upper and lower limb amputation who visited our single-site hospital-based outpatient Physical Medicine and Rehabilitation clinic from January 2018 to January 2019 and who met the study inclusion criteria. This clinic serves the largest volume of patients in the area who require post-amputation rehabilitation and prosthetic management.

The inclusion criteria were that the subjects should be patients who had undergone unilateral, bilateral, or multiple upper and lower limb amputation performed at or above the wrist for the upper limb and at or above the ankle for the lower limb and be 20 years or older at the first visit during the study period. Data were collected only from the first visit during the study period in case the patient had a subsequent follow-up visit. In addition, amputations from all etiologies (trauma, disease-related, neoplasm, and congenital) were included. The exclusion criteria were patients with major amputation younger than 20 years old. Other than education level, data obtained from the electronic medical record included age, gender, race, etiology of amputation, laterality of amputation, level of amputation, and K level. All data were gathered on a standardized form. The education level was divided into five separate categories: below high school, high school, some college, college, and an advanced college degree. Below high school was designated to subjects who attended any level of elementary, middle, or high school but did not graduate. High school was designated to subjects who graduated high school or equivalent. Some college was designated to subjects who attended college but did not graduate from the program they had enrolled in. College was designated to subjects who completed the program they enrolled in but did not receive bachelor, master, or PhD degree. Advanced college degree was categorized as any subject who graduated with bachelor, master, and PhD degree.

Descriptive statistics were utilized to examine the demographic and clinical data. All variables were calculated as a percentage from the sample. Age was also presented as range and average.

## Results

During the one-year study period, 127 subjects who met the study inclusion criteria were included in the study. Descriptive statistics of the demographic and clinical variables are presented in Table 1.

**Table 1:** Background demographic and clinical characteristics of the study population BL, bilateral; MLA, multiple limb amputation; WD, wrist disarticulation; TH, transhumeral amputation; AD, ankle disarticulation or Syme’s amputation; BKA, below the knee amputation; KD, knee disarticulation; AKA, above the knee amputation; HD, hip disarticulation

Study Variables	n (%)
Age	
<40	20 (15.75%)
41-50	24 (18.90%)
51-60	32 (25.20%)
>60	51 (40.16%)
Gender	
Male	84 (66.1%)
Female	43 (33.9%)
Race	
African-American	77 (60.6%)
Caucasian	50 (39.4%)
Etiology of Amputation	
Disease-related	95 (74.8%)
Trauma	28 (22%)
Neoplasm	2 (1.6%)
Congenital	2 (1.6%)
Laterality	
Unilateral	103 (81.1%)
BL/MLA	24 (18.9%)
Level of Amputation	
WD	1 (0.8%)
TH	1 (0.8%)
AD	1 (0.8%)
BKA	66 (51.9%)
KD	2 (1.6%)
AKA	31 (24.4%)
HD	1 (0.8%)
BL/MLA	24 (18.9%)
Functional Level	
K-0	3 (2.4%)
K-1	25 (19.7%)
K-2	37 (29.1%)
K-3	60 (47.2%)
K-4	0 (0%)
N/A	2 (1.6%)

The average age of the subjects in this study was 55.9 years (range 21-91). Of these, 51 (40.16%) were older than 60 years, followed by 32 (25.20%) 51-60 years old, 24 (18.90%) 41-50 years old, and 20 (15.75%) under 40 years old. Within this sample, 84 (66.1%) were men and 43 (33.9%) were women. The majority of the sample, 77 (60.6%) were African-American with the rest of the sample 50 (39.4%) Caucasian. Most of the subjects, 95 (74.8%) had amputation secondary to a disease (PAD, non-healing diabetic ulcer, infection, etc.) followed by 28 (22%) trauma, two (1.6%) congenital malformation, and two (1.6%) neoplasm. Most of the subjects, 103 (81.1%) had unilateral amputation with the rest of the 24 (18.9%) bilateral or multiple limb amputation. Level of amputation was distributed as 66 (51.9%) below the knee, 31 (24.4%) above the knee, two (1.6%) knee disarticulation, one (0.8%) hip disarticulation, one (0.8%) wrist disarticulation, one (0.8%) transhumeral, one (0.8%) ankle disarticulation (Syme’s amputation), and 24 (18.9%) bilateral/multiple limb amputation. Functional level was distributed as 60 (47.2%) K-3, 37 (29.1%) K-2, 25 (19.7%) K-1, three (2.4%) K-0, two (1.6%) N/A, and zero (0%) K-4. Finally, the majority of the subjects in this study, 43 (33.8%) had high school education, followed by 33 (26%) with below high school education, 25 (19.7%) with a college education, 18 (14.2%) with some college education, and eight (6.3%) with advanced college degree education (Table 2).

**Table 2:** Distribution of education level among patients with major limb amputation

Education Level	n (%)
Below high school	33 (26%)
High school	43 (33.8%)
Some college	18 (14.2%)
College	25 (19.7%)
Advanced college degree	8 (6.3%)

## Discussion

The present study is the first to collect education level data on patients with major limb amputation in parts of the Pee Dee region of South Carolina. Previous studies have reported education level as part of their demographic data collection. Sinha et al. found that 71% of amputees had high school academic training, 9% had a university education, and 18% had no schooling [2]. Nunes et al. found that 65% of amputees had a low level of education (subjects had not completed primary education) and 35% had a high level of education [14]. Raichle et al. reported that 5.5% of their lower limb amputee subjects had 9th grade or less education, 6.5% had 10th to 11th grade education, 19.1% had high school education, 10.2% had vocational/technical school education, 29.1% had some college education, 17.8% were college graduate, and 11.6% had graduate school education [4]. Corey et al. showed that 56.3% completed high school and 43.7% did not complete high school [16]. Lastly, Ephraim et al. found that 6.2% had less than high school education, 26.6% graduated high school, and 67.2% had greater than high school education [1]. However, due to the methodological differences in the education level data collection, it is difficult to completely compare the available studies. Nonetheless, many of the previously published studies have associated lower education level not only with an increased incidence of amputation, but also with worse outcomes after amputation. 

The results from our study demonstrated that a little over one-fourth of the sample (26%) had below high school education. This is significantly higher compared to the overall general population in the state of South Carolina (12.9%). It is also higher compared to the general population of Florence county (15.4%), Darlington county (17.9%), Marion county (19.2%), Marlboro county (24.1%), and Dillon county (25.1%) [17]. Furthermore, only 6.3% of our sample had advanced college degree which is lower compared to the state of South Carolina (27.4%), Florence county (23.1%), Darlington county (17.2%), Marion county (14.4%), Marlboro county (9.4%), and Dillon county (11.1%) [17].

Association between health and education has been established and numerous mechanisms through which education influences health has been suggested [18]. The most prominent mediating mechanisms can be grouped into four categories: economic, health-behavioral, social-psychological, and access to health care. Education may determine and affect individuals’ knowledge, reasoning, healthier lifestyles, income, successful long-term marriage, social support, and access to better healthcare, all of which protect or enhance health. Less-educated adults reported worse general health, more chronic conditions, and more functional limitations and disability [18]. Similarly, our study showed lower education level of patients with major limb amputation compared to the general population, which may suggest that barriers such as the ability to understand basic health information, false health beliefs, disease self-management and compliance with treatment, and limited financial recourses may be an issue when dealing with patients with major limb amputation in our region. Furthermore, lower education level has been found to be a predictive factor for higher no-show rates and adherence to treatment [19,20]. Although not the aim of this study, we have also noticed a high no-show rate among amputee patients in our clinic. All of that may result in delayed medical care, rehabilitation, prosthetic fitting, and gait training. Delayed care may also increase the chance of developing complications such as joint contractures, muscle weakness, deconditioning, and residual limb ulcerations. For example, time from amputation to gait retraining has been shown to be predictive of prosthetic non-use with increased time inversely related to the likelihood of ambulation with a prosthesis [15,21]. In addition, high no-show rates may affect the cost of care and resource planning [22]. 

Our study also has some limitations. The study was limited by the nature of a single-clinic retrospective chart review. However, a standardized template for routine collection of amputee pertinent information has been implemented in our amputee clinic prior to this study, which has captured all of the data used in this study and has eliminated the problem of missing data. Only outpatient amputee subjects were included. This may have affected our overall education level distribution. However, these patients are the ones most likely to survive longer, be ambulatory, and require prosthetic care. We also did not investigate the education level distribution by county. This may have possibly given us better comparison and understanding by county. However, we would have needed a larger sample from each individual county, which may be difficult to obtain secondary to the population size and prevalence of patients with major limb amputation in each individual county.

## Conclusions

In conclusion, patients with major limb amputation in Florence and neighboring counties have a lower education level compared to the general population in the region and the state of South Carolina. Education level as a part of the broader and more complex socioeconomic status may be a possible barrier in the process of post-amputation rehabilitation and long-term care of patients with major limb amputation. More aggressive approaches such as closer follow-ups, patient education and improving health literacy, confirmation that patients understand treatment recommendations, and more accessible transportation are some of the steps which may possibly improve the outcomes and the wellbeing of this population.
